# The impact of prebiotics, probiotics and synbiotics on the prevention and treatment of atopic dermatitis in children: an umbrella meta-analysis

**DOI:** 10.3389/fped.2025.1498965

**Published:** 2025-03-21

**Authors:** Lifeng Wang, Lijuan Xu

**Affiliations:** Department of Dermatology, Beijing Luhe Hospital, Capital Medical University, Beijing, China

**Keywords:** probiotics, synbiotics, atopic dermatitis, children, umbrella-meta-analysis

## Abstract

**Background:**

Studies have suggested that the administration of prebiotics, probiotics and synbiotics (pre-, pro-, and synbiotics) may potentially decrease the incidence of atopic dermatitis (AD) and alleviate its severity in children; however, recent studies have yielded inconclusive findings.

**Objective:**

This umbrella meta-analysis aimed to comprehensively assess the effect of pre-, pro-, and synbiotics on AD among children.

**Methods:**

A systematic search was carried out in the PubMed and Scopus databases up to April 2024 to identify relevant meta-analyses. Relative risks (RR) and weighted mean differences (WMD) along with their 95% confidence intervals (CI) were pooled using a random effects model to evaluate the impacts on both the incidence of AD and its severity, as assessed by the Scoring Atopic Dermatitis (SCORAD) index.

**Results:**

This umbrella meta-analysis included 38 meta-analyses, with 127,150 participants. The analysis suggested that intervention with pre-, pro-, and synbiotics significantly reduced the incidence of AD (RR = 0.74, 95% CI: 0.70–0.79), which was confirmed by subgroup analyses. The treatment significantly reduced SCORAD score (WMD = −3.75, 95% CI: −5.08 to −2.42). In subgroup analysis, multi-strain probiotics, *Lactobacillus*, synbiotics, and pre-, pro-, and synbiotics mixtures were found to significantly decrease the SCORAD score, while, *Bifidobacterium* and prebiotics alone did not show a significant effect on the SCORAD score. The treatment resulted in a significant decrease in SCORAD score among children with moderate to severe AD, but not in subjects with mild AD.

**Conclusions:**

Probiotics and synbiotics could be promising interventions to reduce the risk of developing AD and alleviate its severity in children.

## Introduction

Atopic dermatitis (AD) is a prevalent chronic inflammatory skin disease typically commencing during childhood, marked by itching and recurring eczematous lesions (1). The global prevalence of atopic AD has been on the rise in recent decades, impacting as many as 20% of children (2). Around 60% of individuals with AD experience onset before reaching 1 year of age, with 85% developing the condition before the age of 5; additionally, nearly a quarter of children diagnosed with AD may carry the condition into their young adult years (3). AD can substantially affect the children's quality of life and commonly heightens the susceptibility to asthma, allergies, and mental health complications (1, 4). The etiology of AD is multifaceted, arising from intricate interplays among skin barrier impairment, immunological responses, genetic predispositions, and environmental influences (5, 6). The primary treatment approach presently encompasses topical corticosteroids, antihistamines, and in some cases, antibiotics, although prolonged medication usage may result in undesired side effects (7). Nevertheless, these therapies frequently prove inadequate for addressing moderate to severe cases of AD, with symptoms prone to rapid recurrence following treatment cessation (3, 8). Considering the widespread occurrence of AD, its potential enduring health implications, and the safety issues related to current AD medications, the pursuit of novel therapies demonstrating both efficacy and safety for both preventive and therapeutic purposes holds considerable merit.

It has been recently identified that alterations in gut microbiome composition play a key role in the development of AD and other allergic diseases (9). Research indicates that an imbalance in these microbiomes can lead to altered immune responses, which are associated with inflammatory skin conditions such as AD, psoriasis, and acne (10). Specifically, gut dysbiosis may precede the onset of AD, suggesting a significant interplay between gut health and skin integrity. The gut microbiome influences skin health through mechanisms such as the production of short-chain fatty acids, which help regulate inflammation and maintain skin barrier function (11). Furthermore, both gut and skin microbiomes interact bidirectionally, meaning that disturbances in one can adversely affect the other, ultimately contributing to the pathogenesis of various skin disorders (12). Understanding these connections is essential for developing targeted therapeutic strategies that address both gut and skin dysbiosis in managing dermatological conditions.

A shift in gut bacterial diversity, characterized by an increase in *Faecalibacterium prausnitzii*, *Clostridium*, and *Escherichia*, alongside decreased *bifidobacteria* species observed in AD patients, could result in the release of molecules capable of harming the intestinal epithelium (12, 13). These alterations impact the skin condition via neuroendocrine, immunological, and metabolic pathways, hypothesized to contribute to the development of AD (14). Accordingly, prebiotics, probiotics, and synbiotics (pre-, pro-, and synbiotics) have emerged as potential interventions to prevent and treat AD in children by modulating the gut microbiome (15). Prebiotics are non-digestible food ingredients that stimulate the growth of beneficial gut bacteria, while probiotics are live microorganisms with health benefits. Synbiotics combine probiotics and prebiotics with the aim of synergistically improving gut microbial composition and function (16). Several meta-analyses have evaluated the effects of pre-, pro-, and synbiotics on AD in children. However, the results have been mixed, with some studies showing benefits (17, 18) and others finding no effect (19, 20). The heterogeneity in study designs, probiotic strains, duration of treatment, age of children, sample size, and outcome measures has made it difficult to draw definitive conclusions. This umbrella meta-analysis aimed to synthesize the evidence from existing meta-analyses to clarify the role of these interventions in preventing and treating AD in children.

## Methods

We followed the Preferred Reporting Items for Systematic Reviews and Meta-Analyses (PRISMA) guidelines to report this umbrella meta-analysis (21).

### Literature search strategy

The PubMed/MEDLINE and Scopus, databases were systematically searched for relevant meta-analyses published up to April 2024 using the following text words and medical subject terms: (Probiotic* OR Prebiotic* OR Synbiotic* OR lactobacillus OR Bifidobacterium OR bifidobacteria OR lactobacilli OR saccharomyces) AND (eczema OR atopic eczema OR atopic dermatitis OR atopy OR sensitization OR allergic OR allergy OR allergies) AND (meta-analysis). The search was restricted to articles published in the English language. Additionally, we conducted manual searches of published reviews and their references to find any additional studies that align with the inclusion criteria.

### Inclusion criteria

All meta-analyses of randomized clinical trials (RCTs) assessing the impact of pre-, pro-, and synbiotics on AD, whether for prevention or treatment of the condition, were incorporated based on the following criteria: (1) target participants aged <18 years; (2) the intervention subjects (pregnant and/or nursing mothers to children) received pre-, pro-, or synbiotics orally; (3) placebo administered to the control group; (4) The outcomes included the risk of AD incidence and changes in the severity of the disease, as measured by the Scoring Atopic Dermatitis (SCORAD) index; (5) AD diagnosis aligned with standard criteria. Studies with irrelevant intervention, animal studies, letters, narrative reviews, protocols, comments, republished studies, and studies that were on the other allergic diseases were excluded. Two independent reviewers conducted eligibility assessments, resolving disagreements through author discussions.

### Data extraction

Two reviewers independently extracted data from each study, utilizing a pre-designed datasheet, who then cross-checked each other's findings to prevent any mistake. The extracted data included, publication year, author name, sample size, number of analyzed studies, intervention details, risk of bias (ROB) assessment, the relative risk (RR) with 95% confidence interval (CI) for the incidence of AD, and weighted mean difference (WMD) with 95% CI for changes in SCORAD score after the treatment with pre-, pro-, and synbiotics, compared to the placebo. Moreover, the results of subgroup analyses based on the age of children, follow-up duration, type of pre-, pro-, and synbiotics, and supplemented subjects (children only, mothers and children, mothers only) we*re extracted*.

### Quality assessment

The AMSTAR 2 (A Measurement Tool to Assess Systematic Reviews 2) was used to evaluate the quality of the included meta-analyses (22). The AMSTAR 2 tool consists of 16 items that cover various aspects of the systematic review process, such as the research question, the inclusion and exclusion criteria, the literature search, the risk of bias assessment, the meta-analysis methods, and the interpretation of the results. Based on the AMSTAR 2, the quality of the studies were categorizes as critically low, low, moderate, and high.

### Statistical analysis

For prevention studies, the RRs with 95% CIs were used as effect size to assess the effect of the intervention on the incidence of AD. For treatment studies, WMDs with their 95% CIs for SCORAD score in the treatment group, compared to the placebo, were applied to pool the data. Pooled estimates were obtained using a random-effects model according to the Der Simonian–Laird approach (23, 24). Heterogeneity across the studies was assessed using the *χ*^2^ test, with the degree of heterogeneity quantitatively evaluated through *I*^2^ (25). An *I*^2^ value exceeding 50% signified a notable level of heterogeneity. Subgroup analyses based on the quality of studies, treatment duration, children's age, type of intervention, and supplemented subjects (children only, mothers and children, mothers only) were carried out to identify the sources of the heterogeneity. Potential publication bias was evaluated using funnel plots and Egger's linear regression. If significant evidence of publication bias was identified, the pooled effect size was adjusted for the observed bias using the trim-and-fill method (26). Sensitivity analysis was conducted by systematically removing individual studies from the primary analyses to assess whether the pooled estimates were influenced by any particular study. The STATA version 14 (STATA Corporation, College Station, TX, USA) was applied to conduct all tests.

## Results

### Basic characteristics of the eligible studies

**Figure 1:**
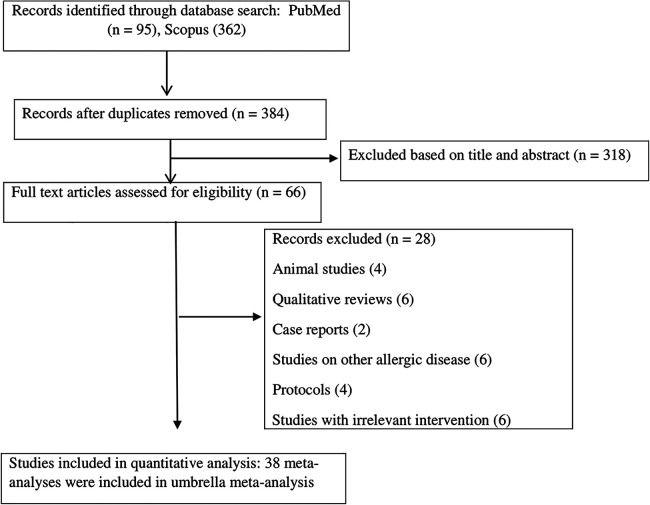
Flow diagram for the study selection.

Initially, a total of 357 studies was collected, comprising 95 articles from PubMed and 362 from the Scopus database. After removing duplicate articles (73 studies) and excluding those that did not align with the research topic following title and abstract reviews (318 studies), an additional 28 irrelevant studies were excluded during full-text screening because they were qualitative reviews, research protocols, animal studies, case reports, studies on other allergic disease, or had an irrelevant intervention. Ultimately, this umbrella meta-analysis incorporated 38 articles (1–4, 8, 17–20, 27–55) published between 2007 and 2023, comprising a total of 127,150 participants. The literature screening process is illustrated in Figure 1. Twenty-five studies (113,083 participants) examined the risk of AD incidence (1, 3, 17–20, 29–33, 35–38, 41, 44–46, 48–53), while 15 studies (24,719 participants) assessed the severity of AD using the SCORAD index (2, 4, 8, 18, 27, 28, 34, 39, 40, 42, 43, 47, 52, 54, 55). The sample size of the included studies varied from 242 to 31,252 subjects. The risk of bias (ROB) in the primary studies was evaluated using different tools such as the Cochrane ROB tool, Jadad score, and PEDro tool. The percentage of primary studies with low ROB in each meta-analysis displayed significant variability, spanning from 0% to 100%. The majority of the included studies, performed subgroup analyses based on the type of intervention and supplemented population; for such studies, in addition to the main analysis, we used the results of the various subgroups to re-analyze the information by pooling the data from different studies. Data on the impact of multistrain probiotics were available in 30 studies, *Lactobacillus* in 20 studies, *Bifidobacterium* in 10 studies, and the combined effect of prebiotics and probiotics (synbiotics) in 5 studies among the analyzed studies. Concerning the supplemented population, the analysis involved 27 studies with children only as the intervention subjects [timing: started from birth to 13 years of age; median follow-up: 10 months (range: 4–54 months)], 20 studies with pregnant mothers and children as the intervention subjects [timing: started from 24 weeks before delivery (mother) to 12 months of age (infants); median follow-up: 3.5 years (range: 1–7 years)], 13 studies with pregnant mothers only [timing: started from 2 to 8 weeks before delivery until delivery; median follow-up: 4 years (range: 1–7 years)], 2 studies with pregnant mothers and breastfeeding mothers [Timing: started from the first trimester of pregnancy to the end of breastfeeding, median follow-up: 4 years (range: 2–6 years)], and 2 studies with pregnant mothers, breastfeeding mothers, and children as the intervention subjects [Timing: mothers started 4–8 weeks before delivery to the end of breastfeeding, infants started from birth to 12 months of age; median follow-up: 2.5 years (range: 1–5.5 years)]. The most commonly used genus of probiotics were *Lactobacillus* and *Bifidobacterium*. The most commonly used species and strains of probiotics were *L. rhamnosus GG*, *Lactobacillus acidophilus AD031*, *L. reuteri ATCC 57730*, *Lactobacillus rhamnosus HN001*, *Lactobacillus plantarum CJLP133*, *Lactobacillus fermentum GM090*, *Lactobacillus salivarius LDR0723*, *Lactobacillus casei CECT 9104*, *Bifidobacterium lactis W52*, *Bifidobacterium animalis subsp. lactis Bb-12*, *Bifidobacterium longum BL999*, *Bifidobacterium bifidum BGN4*, and *Bifidobacterium breve Bb99*.

Based on the AMSTAR2 criteria, the methodological quality of the included studies was high in 11 studies, moderate in 20 studies, and low in 7 studies (Supplementary Table S1). Table 1 presents the basic characteristics of the studies that were included in the analysis.

**Table 1:** Characteristics of studies included in the umbrella meta-analysis.

Study	Year	Country	Included studies	Sample size	Risk of bias assessment, high quality/total studies	Intervention type in the main analyses subgroup analyses based on the intervention	Type of probiotics	Supplemented population	Outcomes	Quality
Zuccotti	2015	Italy	17	939	Cochrane tool, 8/17	Multistrain probiotics/*Lactobacillus* *Bifidobacterium*	*Lactobacillus rhamnosus HN001*, *Bifidobacterium animalis subsp lactis HN019*, *L. reuteri ATCC 57730*, *L. rhamnosus LPR*, *Bifidobacterium longum BL999*, *Lactobacillus paracasei ST11 and B. longum BL999*, *Lactobacillus GG*, *Bifidobacterium bifidum BGN4*, *Bifidobacterium lactis AD011*, *Lactobacillus acidophilus AD031*, *Bifidobacterium bifidum W23*, *Bifidobacterium lactis W52*, *Lactobacillus lactis W58*	Pregnant mother & Child	Incidence of AD	Moderate
Garcia-Larse	2017	USA	38	10,947	Cochrane tool, 27/38	Synbiotics/Prebiotics	NR	Pregnant mother & Child Child only	Incidence of AD	High
Zhao	2018	China	8	741	Cochrane tool, 5\8	Multistrain probiotics/*Lactobacillus* *Bifidobacterium*	*Lactobacillus GG*, *Bifidobacterium lactis*, *Lactobacillus fermentum VRI-003 PCC*, *Lactobacillus paracaseii*, *Bifidobacterium bifidum*	Child only	SCORAD score	Low
Panduru	2014	Romania	16	3,495	NR	Multistrain probiotics/*Lactobacillus*	*Lactobacillus GG*, *Bifidobacterium bifidum*, *B lactis*, *Lactococus lactis*, *Lactobacillus rhamnosus*, *Bifidobacterium longum*, *Lactobacillus F 19*, *Lactobacillus acidophilus*, *Bifidobacterium animalis subsp. lactis Bb-12*	Pregnant mother & Child	Incidence of AD	Moderate
Szajewska	2018	Poland	7	889	Cochrane tool, 4/7	*Lactobacillus*	*Lactobacillus rhamnosus GG*	Pregnant mother & Child	Incidence of AD	High
Sun	2021	China	9	2,093	Cochrane tool, 8/9	Multistrain probiotics	*Lactobacillus salivarius*, *L. paracasei*, *Bifidobacterium animalis subspecies lactis*, *Bifidobacterium bifidum*, *LGG*, *Lactobacillus acidophilus*, *B. bifidum*, *B. longum,*	Pregnant mother & Child Child only Pregnant mother only	Incidence of AD	Moderate
Wang	2023	China	37	2,986	Cochrane tool, 6/37	Multistrain probiotics/*Lactobacillus* *Bifidobacterium*	*Lactobacillus salivarius*, *Lactobacillus paracasei*, *Bifidobacterium animalis subsp Lactobacillus and Bifidobacterium bifidum*, *Lactobacillus rhamnosus*	Pregnant mother & Child Child only Pregnant mother only	Incidence of AD	Low
Pelucchi	2011	Italy	18	4,160	Cochrane tool, 7/18	Multistrain probiotics	*L. rhamnosus GG*, *Bifidobacterium animalis subsp. lactis HN019*, *Bifidobacterium bifidum BGN4*, *B. lactis AD011*, *L. acidophilus AD031*, *Lactobacillus lactis W58*, *B. bifidum W23*, *Bifidobacterium lactis W52*, *Bifidobacteria longum BL999*, *L. rhamnosus LPR*, *Lactobacillus paracasei F19*	Pregnant mother & Child Child only Pregnant mother only	Incidence of AD	High
Michail	2008	USA	10	678	NR	Multistrain probiotics/*Lactobacillus*	*LGG*, *B lactis Bb-12*, *L rhamnosus 19070-2*, *L reuteri DSM 122460*, *L rhamnosus LC705*, *B breve Bbi99*, *Propionibacterium freudenreichii ssp shermani JS*, *L fermentum VRI-003 PCC*	Child only	SCORAD score	Low
Li	2018	China	28	6,907	Jadad score, 24/28	Multistrain probiotics/*Lactobacillus* *Bifidobacterium*	*LGG*, *B. breve 12*, *L. rhamnosus LC705*, *B. breve Bb99*, *L. reuteri ATCC 55730*, *L. rhamnosus HN001*, *B. lactis HN019*, *B. longum 999*, *L. paracasei F19*, *B. bifdum*, *B. lactis*, *L. lactis*, *B. bifdum BGN4*, *L. acidophilus*, *L. salivarius CUL61*, *B. longum BB536*	Pregnant mother & Child Child only Pregnant mother only	Incidence of AD	Moderate
Kim	2014	South korea	25	1,599	Jadad score, 22/25	Multistrain probiotics/Synbiotics *Lactobacillus* *Bifidobacterium*	*Bifidobacteria breve*, *Bifidobacterium bifidum*, *Bifidobacteria lactis*, *Lactobacillus acidophilus*, *Lactobacillus casei*, *Lactobacillus fermentum*, *Lactobacillus rhamnosus*, *Lactobacillus GG*, *Lactobacillus helveticus*, *Lactobacillus paracasei*, *Lactobacillus plantarum*, *Lactobacillus rhamnosus*, *Lactobacillus reuteri*, *Lactobacillus sakei*, *Lactobacillus salivarius*	Child only	SCORAD score	Moderate
Huang	2017	China	13	1,070	Cochrane tool, 11/13	Multistrain probiotics	*Lactobacillus rhamnosus strain GG*, *Lactobacillus paracasei*, *Lactobacillus fermentum*, *B. bifidobacterium infantis*	Child only	SCORAD score	Moderate
Doege	2011	Germany	7	2,843	Cochrane tool, 2/7	Multistrain probiotics/*Lactobacillus*	*Lactobacillus GG*, *L. rhamnosus LC705*, *Bifidobacterium breve Bb99*, *Propionibacterium freudenreichii ssp. Shermanii*, *L. rhamnosus HN001*, *B. animalis ssp. lactis HN019*, *Bifidobacterium lactis Bb12*, *L. reuteri*	Pregnant mother only	Incidence of AD	Low
Dang	2013	China	14	2,550	NR	Pre-, pro- and synbiotics/Synbiotics Prebiotics *Lactobacillus* *Bifidobacterium*	*Lactobacillus paracasei*, *Lactobacillus rhamnosus*, *Bifidobacterium longum BL999*, *L. rhamnosus GG*, *Bifidobacterium animalis subsp. lactis Bb-12*, *Lactobaccillus acidophilus La-5*, *Bifidobacterium bifidum BGN4*, *B. lactis AD011*, *L. acidophilus*, *Lactobacillus rhamnosus HN001*, *B. animalis subsp lactis strain HN019*, *Lactobacillus reuteri ATCC*, *L. acidophilus*, *L. rhamnosus LC705*, *Bifidobacterium breve Bb99*, *Propionibacterium freudenreichii ssp. shermanii JS*, *L. paracasei ssp. paracasei strain LF19*, *Bifidobacterium lactis Bb12; B. bifidum W23; B. lactis W52*	Pregnant mother & Child	Incidence of AD	Moderate
Xue	2023	China	9	1,000	Cochrane tool, 2/9	Multistrain probiotics	*Lactobacillus rhamnosus GG*, *Lactobacillus reuteri*, *Bifdobacterium infantis*, *Lactobacillus paracasei CBA L74*, *Lactobacillus acidophilus L-92*, *Bifdobacterium animalis subsp lactis LKM512*, *Lactobacillus paracasei K71*, *Bifdobacterium lactis CECT 8145*, *B Longum CECT 7347*, *and Lactobacillus casei CECT 9104*	Child only	SCORAD score	Moderate
Chang	2016	China	6	369	Cochrane tool, 3/6	Synbiotics/Prebiotics	*Lactobacillus rhamnosus*, *Lactobacillus acidophilus DDS-1*, *Bifidobacterium lactis UABLA-12*, *Bifidobacterium breve*, *Lactobacillus casei*, *Streptococcus thermophilus*, *Bifidobacterium infantis*, *Lactobacillus bulgaricus*, *Lactobacillus salivarius*, *L rhamnosus GG (ATCC 53103) and LC705 (DSM 7061)*, *Propionibacterium freudenreichii subsp shermanii JS*, *Bifidobacterium longum subsp infantis M63*	Child only	SCORAD score	Modrate
Tanojo	2023	Indonesia	3	242	Cochrane tool, 1/3	Multistrain probiotics	*L. paracasei CBA L74*, *L. rhamnosus*, *L. sakei*, *L. acidophilus L-92*	Child only	SCORAD score	Low
Lopez	2021	Ecuador	17	1,252	Cochrane tool, 13/17	Multistrain probiotics/*Lactobacillus*	*Bifidobacterium lactis CECT 8145*, *B longum CECT 7347*, *Lactobacillus casei CECT 9104*, *Lactobacillus plantarum IS-10506*, *Lactobacillus paracasei GMNL-133*, *Lactobacillus fermentum GM090*, *Lactobacillus rhamnosus GG*, *Lactobacillus plantarum CJLP133*, *Lactobacillus paracasei CNCM I-2116*, *Bifidobacterium lactis CNCM I-3446*, *Lactobacillus fermentum VRI-033 PCC*, *Bifidobacterium lactis*, *Lactobacillus rhamnosus (19070-2) and Lactobacillus reuteri (DSM 122460)*	Child only	SCORAD score	Low
Jiang	2020	China	16	3,049	Cochrane tool, 14/16	Multistrain probiotics	*Lactobacillus rhamnosus HN001*, *Bifdobacterium animalis subsp. lactis HN019*, *Bifdobacterium bifdum BGN4 + Bifdobacterium lactis AD011* *+* *Lactobacillus acidophilus AD031*, *L. rhamnosus ATCC*, *L. acidophilus LAVRI-A1*, *L. rhamnosus GG*, *L. acidophilus La-5_x005f*, *B. animalis*, *Lactobacillus salivarius CUL61 + Lactobacillus paracasei CUL08*, *B. animalis subsp. lactis CUL34*, *B. bifdum CUL20*, *B. bifdum W23*, *B. lactis W52*, *Lc. lactis W58*, *Bifdobacterium infantis*, *Streptococcus thermophilus*, *Lactobacillus sakei KCTC 10755BP*, *B. longum CECT 7347+ Lactobacillus casei CECT 9104*, *Lactobacillus plantarum CJLP133*, *Lactobacillus fermentum VRI-033 PCC*, *Lactobacillus GG (LGG) or MIX [LGG*, *L. rhamnosus LC705 (LC705)]*, *Bifdobacterium breve Bbi99*, *Propionibacterium freudenreichii ssp. shermanii JS*	Pregnant mother & Child Child only Pregnant mother only	Incidence of AD	High
Amalia	2019	Australia	21	31,252	PEDro tool/NR	Multistrain probiotics/*Lactobacillus*	*L. paracasei F19*, *L. rhamnosus LPR*, *B. longum BL999*, *L. rhamnosus GG (LGG)*, *L. acidophilus La-5*, *B. animalis subsp. lactis Bb-12*, *B longum BL999*, *B. lactis AD011*, *B. bifidum BGN4*, *Lactobacillus acidophilus AD031*, *L. salivarius CUL61*, *L. paracasei CUL08*, *B. animalis subsp. lactis CUL34*, *B. bifidum CUL20*	Pregnant mother & Child Child only Pregnant mother only Pregnant mother & breastfeeding mother& infants Pregnant mothers & breastfeeding mothers	Incidence of AD	Moderate
Kuang	2019	China	18	4,356	Cochrane tool, 12/18	Multistrain probiotics/*Lactobacillus*	*Lactobacillus rhamnosus GG*, *L. rhamnosus LC705*, *Bifidobacterium breve Bbi99*, *Propionibacterium freudenreichii ssp. shermanii JS*, *Bifidobacterium infantis*, *Bifidobacterium bifidum*, *Bifidobacterium longum and Lactobacillus acidophilus*, *B. bifidum W23*, *Bifidobacterium lactis W52*, *L. lactis W58*, *Lactobacillus salivarius CUL61*, *Lactobacillus paracasei CUL08*, *Bifidobacterium animalis subsp. lactis CUL34*, *B. animalis subsp. lactis Bb-12*, *L. acidophilus La-5*, *B. bifidum BGN4*, *B. lactis AD011*, *L. acidophilus AD031*	Pregnant mother only	Incidence of AD	High
Mansfield	2014	USA	16	2,797	Cochrane tool, 4/16	Multistrain probiotics/*Lactobacillus* *Bifidobacterium*	*L. rhamnosus*, *Lactobacillus reuteri*, *Bifdobacterium breve Bbi99*, *Propionibacterium freudenreichii ssp. shermanii JS*, *Bifidobacterium longum*, *Lactobacillus acidophilus*, *Bifidobacterium animalis subsp. lactis Bb-12*, *Lactobacillus paracasei*, *Bifidobacterium bifidum*, *Bifidobacterium lactis*	Pregnant mother & Child	Incidence of AD	Moderate
Kim	2023	South korea	25	1,382	Cochrane tool, 0/25	Multistrain probiotics/*Bifidobacterium* *Lactobacillus*	*Lactobacillus fermentum*, *Lactobacillus rhamnosus*, *Lactobacillus helveticus*, *Lactobacillus acidophilus*, *Bifdobacterium lactis*, *Bifdobacterium breve*, *Lactobacillus sakei*, *Lactobacillus salivarius*, *Lactobacillus casei*, *Streptococcus thermophilus*, *Lactobacillus plantarum Bifdobacterium infantis*, *Lactobacillus bulgaricus*, *Bifdobacterium bifdum*, *Lactobacillus pentosus*, *Bifdobacterium longum*, *Lactobacillus casei*	Child only	SCORAD score	Moderate
Cuello-Garcia	2015	Jepan	29	3,509	Cochrane tool, NR	Multistrain probiotics	NR	Child only Pregnant mother only	Incidence of AD	High
Xue	2023	China	21	1,230	Cochrane tool, 15/21	Pre-, pro- and synbiotics/*Lactobacillus* Prebiotics Synbiotics	*Lactobacillus rhamnosus GG*, *Lactobacillus reuteri*, *Bifdobacterium infantis*, *Lactobacillus paracasei CBA L74*, *Lactobacillus acidophilus L-92*, *Bifdobacterium animalis subsp lactis LKM512*, *Lactobacillus paracasei K71*, *Bifdobacterium lactis CECT 8145*, *B Longum CECT 7347*, *and Lactobacillus casei CECT 9104*	Child only	SCORAD score	High
Cao	2015	China	6	1,955	Jadad score, NR	Multistrain probiotics	*ATCC53103*, *DSM7061*, *DSM13692*, *DSM7076*, *LAVRI-A1*, *LF19*, *HN001*, *BL999*, *HN019*	Pregnant mother & Child Child only	Incidence of AD	Moderate
Boyle	2009	Australia	12	781	Cochrane tool, 7/12	Multistrain probiotics/*Lactobacillus*	*L. rhamnosus or LGG*, *B. breve M16-V*, *Bb-12*, *L. rhamnosus Lcr35*, *L. rhamnosus 19070-2 and L. reuteri DSM12246*, *B. lactis*, *L. fermentum VR1-003PCC*	Child only	SCORAD score	Moderate
Elazab	2013	USA	25	4,031	Jadad score, 22 25	Multistrain probiotics	*L reuteri*, *Lactobacillus GG*, *Lactobacillus acidophilus*, *B lactis*, *Lactobacillus HN001*	Pregnant mother & Child Child only	Incidence of AD	High
Husein-ElAhmad	2023	Spain	75	8,754	NR	Multistrain probiotics/*Lactobacillus* *Bifidobacterium*	NR	Pregnant mother & Child Child only Pregnant mother only Pregnant mothers & breastfeeding mothers Pregnant mother & breastfeeding mother& infants	Incidence of AD, SCORAD score	Low
Pan	2022	China	8	2,575	Jadad score, 8/8	Multistrain probiotics	*ATCC53103*, *DSM 7061*, *DSM13692*, *DSM7076*, *LAVRI-A1*, *LF19*, *HN001* *+* *HN019*, *BL999* *+* *LPR*, *CUL61*, *CUL08*, *CUL34*, *CUL20*, *LPR*	Pregnant mother & Child	Incidence of AD	Moderate
Voigt	2022	USA	11	5,437	Cochrane tool, 5/11	*Lactobacillus*	*L. rhamnosus*	Pregnant mother & Child	Incidence of AD	Moderate
Sun	2022	China	17	4,011	Cochrane tool, NR	Multistrain probiotics	*Propionibacterium*, *Lactobacillus and Bifidobacterium spacies*	Pregnant mother & Child Child only Pregnant mother only	Incidence of AD	Moderate
Fijan	2023	Slovenia	17	1,124	Cochrane tool, 5/17	Multistrain probiotics	*Lacticaseibacillus rhamnosus GG (LGG)*, *Limosilactobacillus fermentum VRI-033 PCC*, *Latilactobacillus sakei KCTC 10755BP*, *Lacticaseibacillus casei DN-114001*, *Lacticaseibacillus paracasei CNCM I-2116*, *Lactiplantibacillus plantarum CJLP133*, *Limosilactobacillus fermentum*, *Lactiplantibacillus pentosus*	Child only	SCORAD score	Moderate
Chen	2022	China	22	NR	NR	Multistrain probiotics	NR	Pregnant mother & Child Child only Pregnant mother only	Incidence of AD	Moderate
Lee	2008	USA	21	1,898	Jadad score, NR	Multistrain probiotics	*LGG*, *Lactobacillus acidophilus LAVRI-A1*, *Lactobacillus reuteri ATCC 55730*	Pregnant mother & Child	Incidence of AD, SCORAD score	Moderate
Makrgeorgou	2018	UK	39	2,599	Cochrane tool, 21/39	Multistrain probiotics/*Lactobacillus* *Bifidobacterium*	*L rhamnosus*, *L GG*, *Lactobacillus casei LOCK 0900*, *Lactobacillus casei LOCK 08*, *Lactobacillus paracasei LOCK 0919*, *Lactobacillus salivarius LDR0723*, *Streptococcus thermophilus*, *Bifidobacterium breve*, *Lactobacillus acidophilus*, *Bifidobacterium infantis*, *Lactobacillus bulgaricus*, *Lactobacillus salivarius W57*, *Lactobacillus lactis W58*, *Bifidobacterium infantis W52*, *Bifidobacterium lactis W18*, *Bifidobacterium longum W51*, *Bacilus cereus*, *Lactobacillus plantarum CJLP 133*, *Lactobacillus paracasei*, *Streptococcus thermophilus TH-4*, *Bifidobacterium bifidum*	Child only	SCORAD score	High
Osborn	2007	Australia	2	432	Cochrane tool, 0/2	Prebiotics	-	Child only	Incidence of AD	High
Osborn	2013	Australia	4	1,218	Cochrane tool, 0/4	Prebiotics	-	Pregnant mother only	Incidence of AD	High

NR, not reported; SCORAD, scoring atopic dermatitis.

### Results of the umbrella- meta-analysis

#### Effect of pre-, pro-, and synbiotics on AD prevention

**Figure 2:**
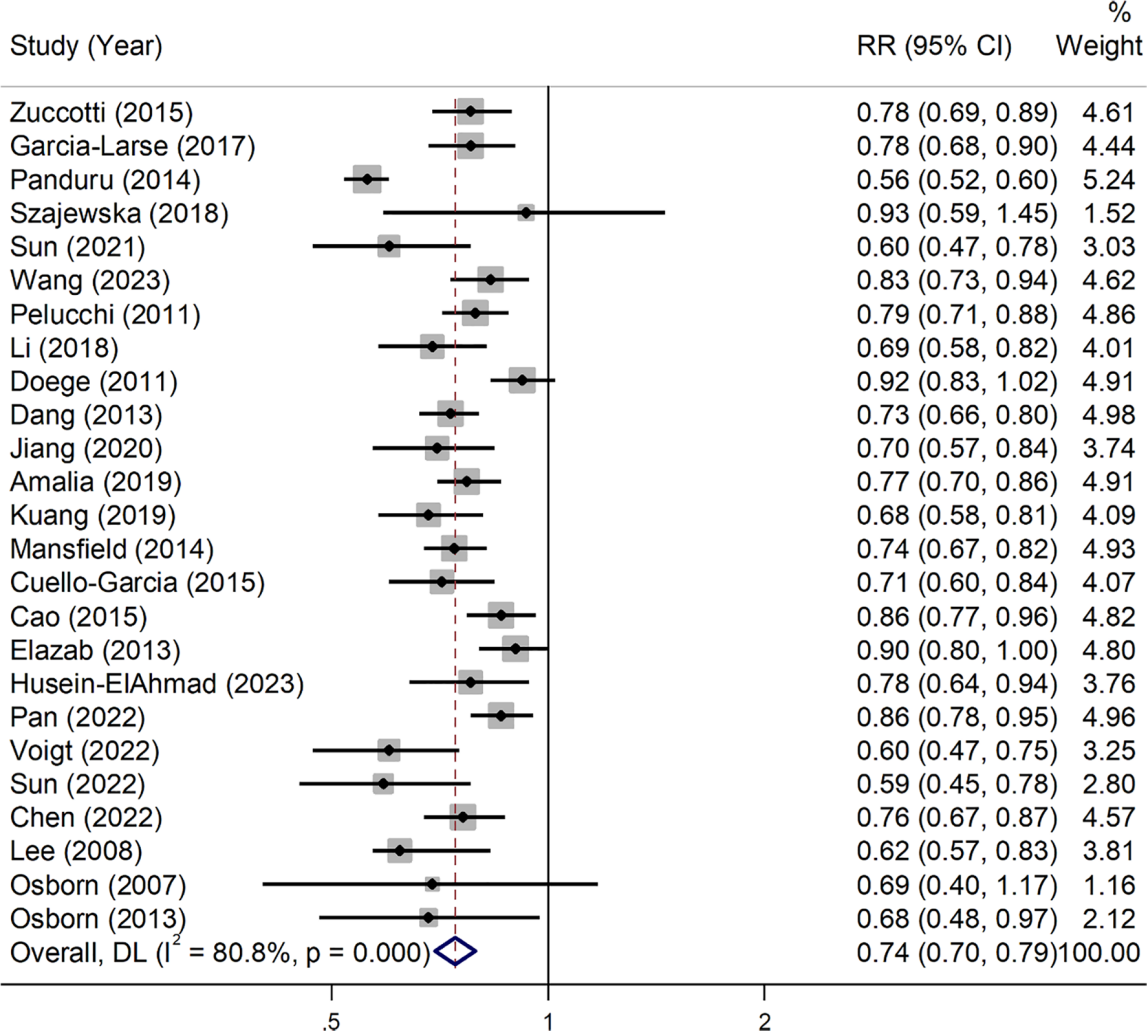
Pooled effects of probiotics on the prevention of atopic dermatitis in children.

Employing the random-effects model, the combined effect sizes indicate that pre-, pro-, and synbiotics intervention has a significant impact on reducing the incidence of AD (RR = 0.74, 95% CI: 0.70–0.79). There was a notable heterogeneity across the analyzed studies (*I*^2^ = 80.8%, *P* = 0.001; Figure 2). This finding was consistently supported by various subgroups according to the quality of studies, type of intervention, supplemented population, children's age, and follow-up duration, with the exception of cases where the duration of supplementation exceeded 12 months (Table 2).

**Table 2:** Subgroup analyses for the effect of probiotics on the prevention of atopic dermatitis in children.

Outcomes	Subgroups	Studies	Test of effect	Test of heterogeneity	
			RR (95% CI)	*I*^2^ (%)	*P*
Quality of studies	Low	3	0.86 (0.78–0.94)	30.9	0.23
	Moderate	13	0.71 (0.65–0.78)	85.4	0.001
	High	9	0.77 (0.71–0.83)	37.1	0.12
Type of intervention	Multi-strain probiotics	19	0.75 (0.69–0.80)	85.0	0.001
	*Lactobacillus*	11	0.78 (0.68–0.89)	65.0	0.006
	*Bifdobacterium*	5	0.75 (0.65–0.87)	35.4	0.18
	Synbiotics	2	0.79 (0.70–0.89)	0.0	0.76
	Prebiotics	4	0.73 (0.61–0.88)	0.0	0.93
	Pre-, pro-, and synbiotics	1	0.73 (0.66–0.80)	-	-
Supplemented population	Pregnant mothers and children Timing: From 24 weeks before delivery (mother) to 12 months of age (infants) Median Follow-up: 3.5 years (Range: 1–7 years)	20	0.74 (0.68–0.79)	80.8	0.001
	Pregnant mothers only Timing: From 2 to 8 weeks before delivery until delivery Median Follow-up: 4 years (Range: 1–7 years)	13	0.70 (0.62–0.79)	60.7	0.003
	Children only Timing: Started from birth to 13 years of age Median Follow-up: 10 months (Range: 4–54 months)	27	0.84 (0.79–0.90)	0.0	0.78
	Pregnant mothers and breastfeeding mothers Timing: From the first trimester of pregnancy to the end of breastfeeding Median Follow-up: 4 years (Range: 2–6 years)	2	0.60 (0.43–0.84)	61.5	0.10
	Pregnant mothers, breastfeeding mothers, and children Timing: Mothers started 4–8 weeks before delivery to the end of breastfeeding, infants started from birth to 12 months of age Median Follow-up: 2.5 years (Range: 1–5.5 years)	2	0.72 (0.61–0.85)	13.8	0.28
Duration of supplementation	≤3 months	2	0.51 (0.30–0.87)	33.4	0.22
	>3 months, ≤6 months	2	0.74 (0.67–0.81)	0.0	0.89
	≤6 months	2	0.65 (0.50–0.86)	0.0	0.82
	>6 months	2	0.78 (0.69–0.88)	0.0	0.32
	>12 months	2	0.93 (0.71–1.22)	50.6	0.15
Age of children	≤1 year	2	0.77 (0.62–0.94)	37.1	0.20
	≤2 years	5	0.69 (0.55–0.87)	74.3	0.004
	>2 years	6	0.72 (0.63–0.83)	47.8	0.08
	≤4 years	2	0.79 (0.68–0.92)	0.0	0.77
	>4 years	1	0.81 (0.67–0.98)	-	-

#### Effect of pre-, pro-, and synbiotics on the severity of AD

**Figure 3:**
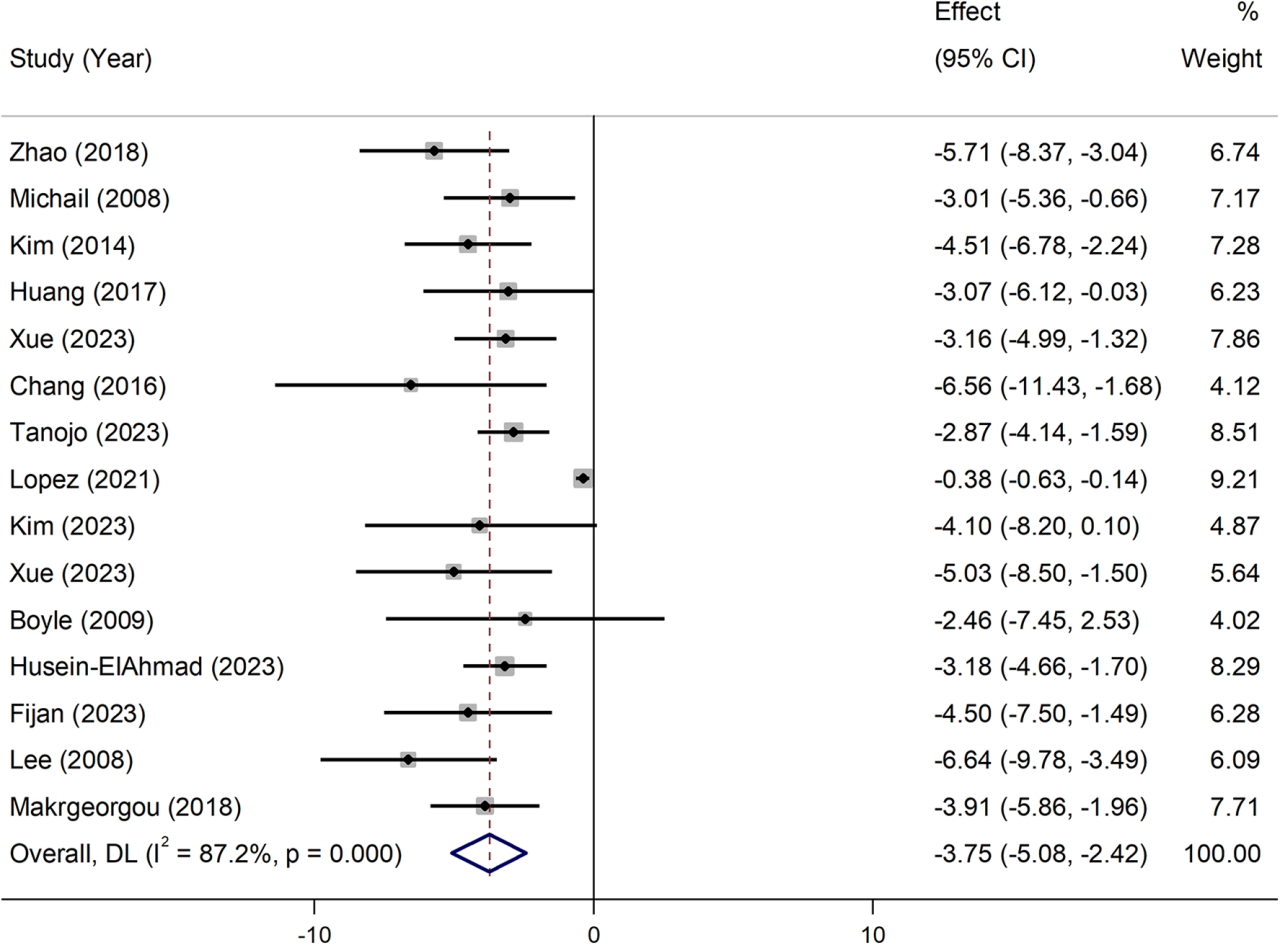
Pooled effects of probiotics on the severity of atopic dermatitis (SCORAD index).

In the overall analysis, pooled effect size from available studies revealed a significant reduction in SCORAD score following the treatment with pre-, pro-, and synbiotics (WMD = −3.75, 95% CI: −5.08 to −2.42), with significant heterogeneity (*I*^2^ = 87.2%, *P* = 0.001; Figure 3). In the subgroup analysis by the type of intervention, treatment with multi-strain probiotics (WMD = −3.34, 95% CI: −4.69 to −1.99), *Lactobacillus* (WMD = −1.79, 95% CI: −3.47 to −0.11), synbiotics (WMD = −7.13, 95% CI: −9.83 to −4.43), and pre-, pro-, and synbiotics mixtures (WMD = −5.05, 95% CI: −8.53 to −1.53) significantly reduced SCORAD score, indicating that the addition of prebiotics to probiotics, yields a more substantial impact compared to the use of probiotics alone. However, no notable effect was observed for *Bifidobacterium* and Prebiotics alone (Table 3). In the subgroup analysis on the severity of disease, the intervention resulted in a significant decrease in SCORAD score among participants diagnosed with moderate to severe AD (WMD = −3.20, 95% CI: −3.55 to −2.84), but not in individuals with mild AD. The observed significant effects were not modified across subgroups based on the quality of studies, supplemented population, supplementation duration, and the age of children (Table 3).

**Table 3:** Subgroup analyses for the effect of probiotics on the severity of atopic dermatitis (SCORAD) in children.

Outcomes	Subgroups	Studies	Test of effect	Test of heterogeneity	
			RR (95% CI)	*I*^2^ (%)	*P*
Quality of studies	Low	5	−2.76 (−4.99 to −0.52)	91.0	0.001
	Moderate	8	−4.20 (−5.25 to −3.16)	0.0	0.53
	High	2	−3.05 (−5.50 to −0.60)	69.1	0.03
Type of intervention	Multi-strain probiotics	12	−3.34 (−4.69 to −1.99)	86.4	0.001
	*Lactobacillus*	9	−1.79 (−3.47 to −0.11)	78.8	0.001
	*Bifdobacterium*	5	−1.24 (−4.00 to 1.52)	77.3	0.001
	Synbiotics	3	−7.13 (−9.83 to −4.43)	0.0	0.52
	Prebiotics	2	−3.57 (−13.595 to 6.81)	91.0	0.001
	Pre-, pro-, and synbiotics	1	−5.05 (−8.53 to −1.53)	-	-
Supplemented population	Pregnant mothers and children Timing: From 24 weeks before delivery (mother) to 12 months of age (infants) Follow-up: Median 3.5 years (Range: 1–7 years)	1	−6.64 (−9.79 to −3.49)	-	-
	Children only Timing: Started from birth to 13 years of age Follow-up: Median 10 months (Range: 4–54 months)	15	−3.37 (−4.62 to −2.12)	84.4	0.001
Duration of supplementation	≤8 months	7	−3.11 (−4.69 to −1.54)	19.6	0.28
	>8 months	7	−2.97 (−3.95 to −2.00)	24.9	0.23
	≤12 months	2	−3.09 (−6.78 to −0.60)	0.0	0.68
	>12 months	2	−4.75 (−7.30 to −2.20)	0.0	0.86
Age of children	≤1 year	6	−1.53 (−2.46 to −0.59)	16.7	0.30
	>1 year	6	−6.38 (−7.07 to −5.69)	4.9	0.38
	≤2 years	2	−0.63 (−3.28 to −0.02)	0.0	0.60
	>2 years	2	−5.79 (−9.19 to −2.38)	0.0	0.62
	≤3 years	1	−0.73 (−11.05 to −1.15)	-	-
	>3 years	1	−2.76 (−4.99 to −0.52)	-	-
Severity of disease	Mild	6	−0.38 (−0.96 to 0.22)	0.0	0.48
	Mild/moderate	2	−1.33 (−1.89 to −0.77)	0.0	0.72
	Moderate/severe	13	−3.20 (−3.55 to −2.84)	0.0	0.58

#### Meta-regression analysis, sensitivity analysis, and publication bias

**Figure 4:**
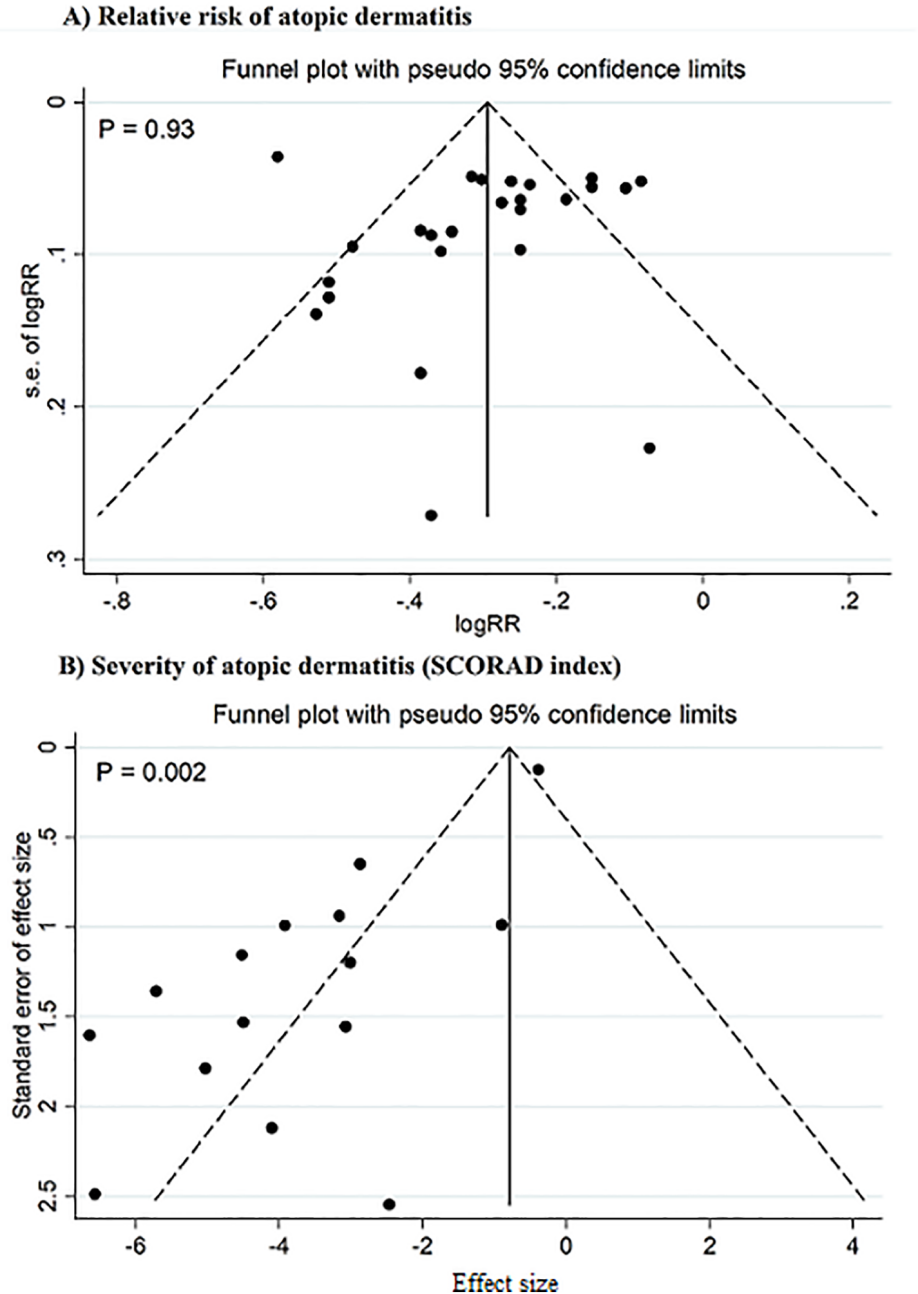
Funnel plot for publication bias. Studies on the relative risk of atopic dermatitis **(A)**, studies on the severity of atopic dermatitis (SCORAD index) **(B)**.

In the meta-regression analysis, the pooled effect sizes were not affected by sample size and the percentage of primary studies with low ROB in each meta-analysis. Sensitivity analysis demonstrated that individual studies did not impact the pooled effect sizes for outcomes. There was no publication bias detected in studies exploring the preventive impact of pre-, pro-, and synbiotics on AD incidence. However, significant evidence of publication bias was noted in studies focusing on the severity of AD (*P* = 0.002; Figure 4). When the pooled effect size was adjusted for the observed bias using the trim-and-fill analysis, the pooled estimate did not change significantly, showing the reliability of the findings. The strength of evidence was low for outcomes (Table 4).

**Table 4:** Strength of evidence.

Outcomes	No of studies	Design	Risk of bias	Inconsistency	Indirectness	Imprecision	Other considerations	Sample size	Relative risk (95% CI)	WMD (95% CI)	Quality	Importance
Risk of atopic dermatitis	25 studies	Meta-analysis of randomized trials	No serious	Serious^b^	No serious	Serious^c^	None	113,083 participants	RR: 0.74 (95% CI: 0.70–0.79)	-	Low	Critical
Severity of atopic dermatitis (SCORAD)	15 studies	Meta-analysis of randomized trials	Serious^a^	Serious^b^	No serious	No serious	None	24,719 participants	-	−3.75 (−5.08 to −2.42)	Low	Critical

CI, confidence interval; WMD, weighted mean difference.

^a^ Significant publication bias.

^b^ Significant heterogeneity.

^c^ Confidence intervals include appreciable harm or benefit.

## Discussion

In this umbrella meta-analysis, we explored the preventive and therapeutic effects of pre-, pro-, and synbiotics on AD in children. The analysis unveiled that both the incidence and severity of AD could be notably diminished through the administration of the intervention to pregnant and/or nursing mothers or to children. Moreover, the analysis of the subgroups identified that probiotics mixtures, *Lactobacillus*, and synbiotics remarkably declined the severity of AD, however, no significant effect was observed for *Bifidobacterium* and prebiotics when administered alone, suggesting that the effects may be strain-specific. The reduction in AD severity was found for children with moderate to severe AD, whereas no such improvement was noted in patients with mild AD.

Recent studies indicated that probiotics influence the immune system's functioning and enhance the intestinal barrier, potentially aiding in the prevention and management of allergic conditions. Newborns are born with a sterile gastrointestinal tract, and the establishment of microflora during the initial postnatal phase plays a role in activating both adaptive and innate immune responses (56). A lack of sufficient microbial stimulation leads to an imbalanced gut microflora, promoting the dominance of a neonatal Th2-driven immune response, thereby contributing to the onset of atopic conditions (57). Research has shown the potential of probiotic supplementation in AD. Nevertheless, a consensus regarding the effectiveness of probiotics for the clinical prevention and treatment of AD is yet to be reached due to controversy in the results of the studies. These discrepancies could be elucidated by variations in children's ages, the timing of probiotic supplementation, intervention objects, duration of follow-up, disease severity, genetic background, and the specific probiotic strains utilized. The current umbrella analysis indicated that the beneficial effects of probiotic/synbiotic interventions in preventing and treating AD in children were comparable when provided during the prenatal and postpartum periods, whether given to mothers or children. Recent studies have demonstrated that the transmission of maternal microbes to offspring begins during pregnancy, creating an initial microbiome in the fetus (38). Microbial DNA has been identified in umbilical cord blood, fetal and placental membranes, amniotic fluid, and meconium (58). Consequently, the intimate immunological interplay between the mother and the fetus allows for the maternal microbiota to impact the immune development of the offspring, potentially influencing patterns of gut colonization in infants and their susceptibility to allergic diseases (59). Since children with AD exhibit distinct gut microbiota profiles in comparison to non-atopic individuals (12, 13), early probiotic supplementation could encourage a more favorable gut microbiota composition that, subsequently, reduces the risk of AD development.

We also revealed that interventions that included synbiotics were more effective in reducing the severity of AD, compared to probiotics alone. Synbiotics possess both probiotic and prebiotic effects, theoretically functioning more effectively than either component in isolation due to their synergetic effects in in modulating the gut microbiota and consequently the immune system (39). This finding is supported by some previous evidence, indicating the possible superior efficacy of synbiotics therapy over probiotics (34). Another result was that probiotic/synbiotic treatment lasting more than 12 months did not result in the prevention of AD. This contrasts with earlier research suggesting that prolonged probiotic use was advantageous in AD prevention (17). The discrepancy could be due to the natural course of AD, which may weaken the efficacy of any treatment over an extended period. However, this conclusion was based on the combined results of only 2 studies, indicating that caution is warranted in its interpretation due to the potential lack of statistical power to identify a distinction. Additionally, no advantage was observed in probiotic/synbiotic therapy for children with mild AD. This finding suggests that considering to the severity of AD is important when determining the need for probiotic/synbiotic supplementation in individuals with AD. Nonetheless, additional research is essential to validate the findings of the current study.

The preventive and therapeutic effects of probiotic/synbiotic supplementation on AD could be explained by several mechanisms, including modulation of the gut microbiome, anti-inflammatory effects, immunomodulation, competitive exclusion of pathogens, and improvement of skin barrier function (60). Probiotics and synbiotics can help restore a healthy gut microbiome by increasing the abundance of beneficial bacteria like Lactobacillus species (61). This can strengthen the gut epithelial barrier and reduce intestinal inflammation, which is linked to the development of AD (60). Probiotics and their metabolites, such as short-chain fatty acids (SCFAs), can downregulate pro-inflammatory cytokines and inhibit the production of reactive oxygen species (ROS). This can help attenuate the chronic systemic inflammation that plays a key role in AD pathogenesis (62). Probiotics can modulate the immune system by enhancing the production of regulatory T cells and reducing the activity of effector T cells (63). This can help restore the balance between Th1 and Th2 responses, which is often dysregulated in AD (64). Probiotics can competitively inhibit the adhesion and growth of pathogenic bacteria by producing antimicrobial substances and occupying binding sites on the intestinal epithelium. This can prevent the overgrowth of harmful microbes that may contribute to AD development (65). Probiotics and their metabolites can enhance the expression of tight junction proteins and increase the production of antimicrobial peptides in the skin (66). This can strengthen the skin barrier and reduce the penetration of allergens and irritants, which can trigger AD flare-ups. Different probiotic strains may have varying mechanisms of action. For example, *Lactobacillus* species have been shown to possess specific biological properties, such as the ability to prevent pathogen adhesion to the intestinal epithelium, which may be crucial for reducing bacterial translocation and modulating the inflammatory response (67). However, further research is needed to elucidate the specific mechanisms involved and to determine the optimal probiotic strains and dosages for AD management.

To our understanding, this study represents the first umbrella meta-analysis examining the impact of pre-, pro-, and synbiotics on AD in children. The strength of this analysis lies in the inclusion of a significant number of studies with a substantial sample size and comprehensive subgroup analyses coupled with meta-regression analyses to pinpoint potential sources of heterogeneity. Moreover, we utilized the GRADE assessment to clarify the quality of evidence, providing a transparent and systematic method for crafting evidence summaries and recommendations. This procedure enhances informed decision-making by reducing uncertainty and errors. Some limitations of this study need to be acknowledged. First, a significant evidence of heterogeneity was detected across the studies. Subgroup analysis revealed that the heterogeneity could be partially attributed to the differences in the quality of meta-analyses, type of intervention, and age of children. We applied a random effects analyses to reduce the effect of the heterogeneity on the outcomes. The Egger's test showed a significant evidence of publication bias in studies on SCORAD. We limited the search strategy to articles published in English languages, which could result in the exclusion of studies in other languages. This limitation could potentially affect the comprehensiveness and generalizability of our findings, as relevant research published in other languages may not have been included. However, the results were stable after adjusting the pooled estimates for the publication bias. Another limitation is that this analysis did not assess the long-term safety of the interventions due to lack of sufficient data in included studies, which is important to be evaluated in future studies. Nevertheless, evidence has shown that pre-, pro-, and synbiotics are generally safe in children (68).

In conclusion, our umbrella meta-analysis revealed that providing probiotics and synbiotics to both mothers and children can serve as effective measures in preventing and treating AD in children diagnosed with moderate to severe AD. However, the effectiveness of *Bifidobacterium* and prebiotics in addressing AD was not substantiated by our study. Future investigations should focus on pinpointing the optimal commencement time for probiotic supplementation, taking into account the pivotal role of prenatal provision, as well as determining the most effective dosage and duration of administration to establish an optimal preventive and therapeutic regimen.

## Data Availability

The original contributions presented in the study are included in the article/Supplementary Material, further inquiries can be directed to the corresponding author.
